# Ten simple rules to build genomics capacity for routine laboratory practice in the Global South

**DOI:** 10.1371/journal.pcbi.1014863

**Published:** 2026-10-15

**Authors:** Emilyn Costa Conceição, Abhinav Sharma, Davi Josué Marcon, Layana Rufino Ribeiro, Danna Karen Corrêa dos Santos, Lilian Eduarda de Oliveira Freitas, Alex Brito Souza, Hendor Neves Ribeiro de Jesus, Maria Cristina da Silva Lourenço, Valnete das Graças Dantas Andrade, Marco Aurélio Almeida de Oliveira, Tatyana Costa Amorim Ramos, Ítalo José Mesquita Cavalcante, Arabela Leal e Silva de Melo, Nabila Ismail, Tulio de Oliveira, Robin Mark Warren, Adriana Ayden Ferreira, Theolis Costa Barbosa Bessa, Cristiane Cunha Frota, Philip Noel Suffys, Karla Valéria Batista Lima, REVIGET Consortium

**Affiliations:** 1 South African Medical Research Council Centre for Tuberculosis Research (SAMRC), Division of Molecular Biology and Human Genetics (MBHG), Biomedical Research Institute (BMRI), Faculty of Medicine and Health Sciences (FMHS), Stellenbosch University, Cape Town, South Africa; 2 Centre for Epidemic Response and Innovation (CERI), School of Data Science and Computational Thinking, Stellenbosch University, Stellenbosch, South Africa; 3 Rede Brasileira de Pesquisas em Tuberculose (REDE-TB), Rio de Janeiro, Rio de Janeiro, Brazil; 4 Programa de Pós-graduação em Biologia Parasitária na Amazônia (PPG-BPA), Universidade do Estado do Pará (UEPA), Belém, Pará, Brazil; 5 Seção de Bacteriologia e Micologia (SABMI), Instituto Evandro Chagas (IEC), Ananindeua, Pará, Brazil; 6 Programa de Pós-graduação em Epidemiologia e Vigilância em Saúde (PPGEVS), Instituto Evandro Chagas (IEC), Ananindeua, Pará, Brazil; 7 Laboratório Central do Amazonas (LACEN-AM), Fundação de Vigilância em Saúde do Amazonas Dra. Rosemary Costa Pinto (FVS-RCP), Manaus, Amazonas, Brazil; 8 Instituto Gonçalo Moniz (IMG), Fundação Oswaldo Cruz (FIOCRUZ), Salvador, Bahia, Brazil; 9 Laboratório de Bacteriologia e Bioensaios em Micobactérias, Instituto Nacional de Infectologia Evandro Chagas (INI), Fundação Oswaldo Cruz (FIOCRUZ), Rio de Janeiro, Rio de Janeiro, Brazil; 10 Laboratório Central do Pará (LACEN-PA), Belém, Pará, Brazil; 11 Laboratório Central do Ceará (LACEN-CE), Fortaleza, Ceará, Brazil; 12 Laboratório Central da Bahia (LACEN-BA), Salvador, Bahia, Brazil; 13 Programa de Pós-graduação em Patologia, Universidade Federal do Ceará (UFC), Fortaleza, Ceará, Brazil; 14 Laboratório de Biologia Molecular Aplicada a Micobacterias, Instituto Oswaldo Cruz, Fundação Oswaldo Cruz, Rio de Janeiro, Rio de Janeiro, Brazil; Carnegie Mellon University, UNITED STATES OF AMERICA

## Abstract

Developing sustainable genomics capacity in the Global South requires more than technology transfer; it depends on human-centered approaches based on the workflows and existing expertise of laboratory personnel, institutional collaboration, and long-term integration into health systems. Drawing on lessons learned from regional capacity-building initiatives, this article proposes ten simple rules for implementing and sustaining genomics capacity within public health and clinical laboratory settings. These rules emphasize local context, strategic planning, continuous learning, and equitable collaboration. Together, they provide a roadmap for laboratories aiming to transform genomic technologies from research tools for diagnosis, surveillance and decision-making, into routine instruments for public health in low- and middle-income countries.

## Introduction

Genomic surveillance enables high-resolution tracking of pathogens, early outbreak detection, and real-time monitoring of disease evolution, thereby improving public health interventions and pandemic preparedness [1–3]. Integration of genomics in public health has been pivotal in managing COVID-19, antimicrobial resistance (AMR), and other infectious threats, leading to improved targeted responses and cost-effective interventions [4–6]. Yet, in the Global South, incorporating these tools into laboratory routines remains challenging due to substantial disparities in infrastructure (particularly high upfront costs for sequencing infrastructure and consumables), human resources, and institutional support. However, strategic procurement, resource sharing, and technological innovations are making genomics increasingly cost-effective, transforming initial investments into substantial long-term savings and improved diagnostic capacity [7,8].

Building capacity for genomics requires more than the acquisition of sequencers or training sessions; it entails strengthening systems that integrate infrastructure, people, and decision policies. Effective capacity building fosters regional autonomy, data sovereignty, and equitable participation in global scientific production. This paper proposes practical lessons, categorized as four major themes (Table 1), including the Consolidation of the Genomic Epidemiological Surveillance Network for Tuberculosis (REVIGET) [9], the Genomic Network for Precision Medicine and Surveillance of High-Prevalence Infectious Diseases in Northern and Northeastern Brazil (REVIGEN) and the GenPath Africa initiatives, being the basis of ten simple rules to guide laboratories and public health institutions in establishing sustainable genomic capacity for routine use (Fig 1). These rules are proposed primarily for laboratory managers and technical personnel responsible for day-to-day implementation. In addition, rules 1, 3, and 10 are relevant to institutional leadership, funders, and public-health policymakers, given their role in resourcing and integrating genomics into broader health systems.

**Table 1:** Conceptual framework summarizing the ten simple rules for building genomics capacity in routine laboratory practice in the Global South, organized into four overarching themes.

Theme	Description	Rules
**1. Diagnose, plan, and prepare the groundwork**	Understand the setting, map needs, and align logistics and management before implementation.	1–3
**2. Build skills through co-creative and continuous learning**	Design iterative training cycles, co-develop protocols, and encourage ownership of knowledge.	4–6
**3. Strengthen systems and communities for sustainability**	Address infrastructure, create shared repositories, and foster communities and champions who sustain learning.	7–9
**4. Integrate genomics into health systems and data ecosystems for sustainability**	Link genomic data with clinical and epidemiological systems for actionable insights.	10

Rules 1–3 focus on preparatory diagnosis, infrastructure, and logistics. Rules 4–6 concentrate on the pedagogical process of building and transferring skills. Rules 7–9 address the social and infrastructural structures that sustain those skills over time. Rule 10 addresses the integration of genomic data into broader health information systems. While several rules touch on related activities, each theme requires distinct actions and stakeholders.

**Fig 1:**
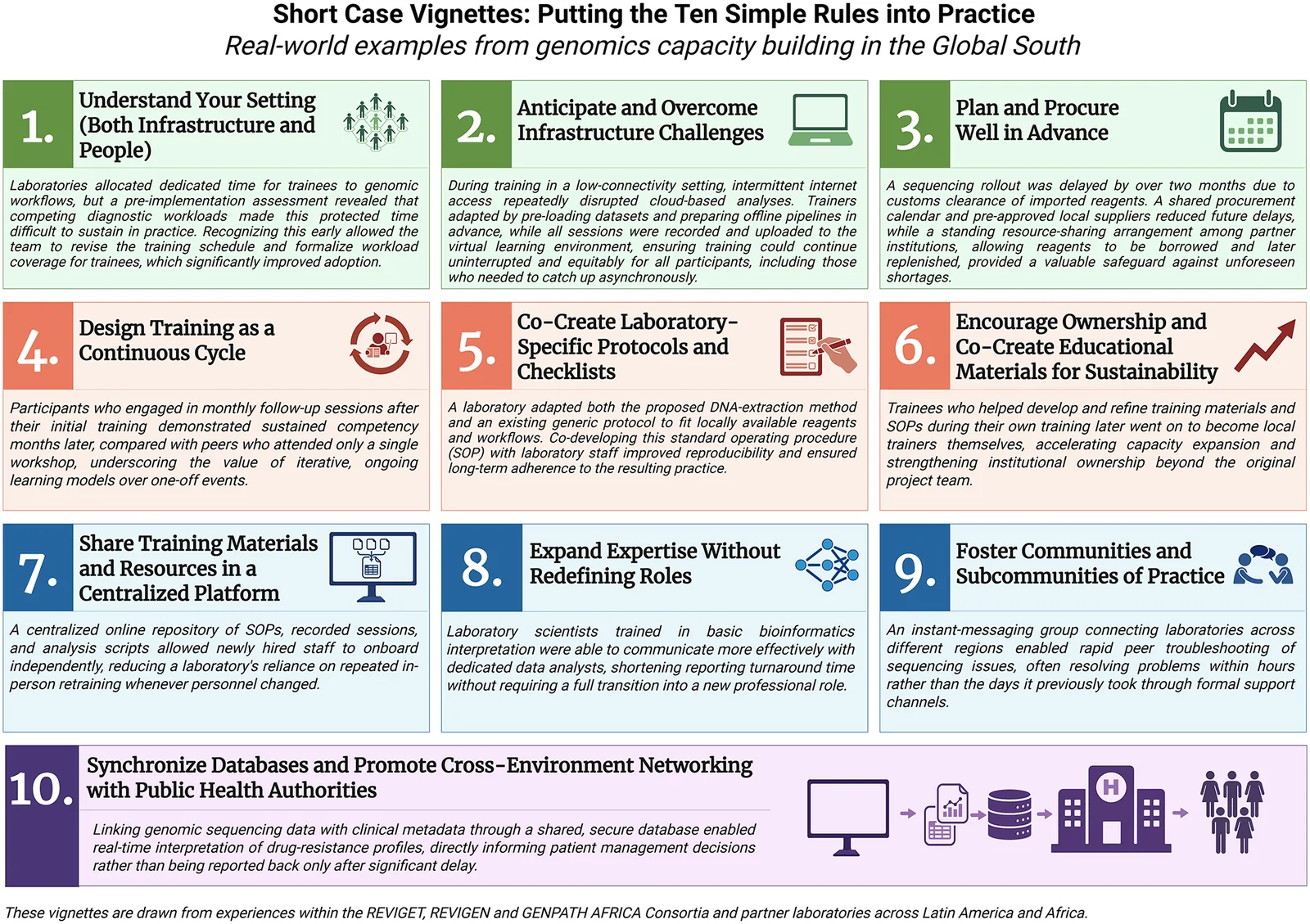
Summary of the ten rules across four principal themes, together with short, anonymised case vignettes illustrating how each rule was applied in practice within the REVIGET, REVIGEN and GenPath Africa initiatives. Created in BioRender. Lima, K. (2026) https://BioRender.com/f7vfwwu.

While the examples stem from public health laboratories in the Global South (Fig 1), the principles are broadly applicable to any setting seeking to integrate genomics into diagnostic and surveillance systems.

### Rule 1: Understand your setting (both infrastructure and people)

Successful capacity building begins with a comprehensive analysis of the local context. Before launching any training or infrastructure investments, laboratories should assess their physical space, biosafety level, sequencing and computational resources, human capital, and institutional priorities. This process should include site visits, listening to the management team and mapping of capacities and bottlenecks to ensure that the next steps are realistic and aligned with on-site needs.

Understanding the human dimension is equally important, identifying those who will perform, interpret, and manage genomic data. Assessing role players, time availability, and motivations ensures that training is relevant and that skills can be applied effectively. The assessment should also align with national public-health priorities, ensuring that laboratory efforts support priority diseases, surveillance goals, and policies defined by health authorities. Aligning capacity building with national programs for pandemic preparedness, tuberculosis control and/or antimicrobial-resistance surveillance, maximizes impact and institutional buy-in.

Engaging institutional leadership for embedding genomics as a public-health priority ensures that management structures support its long-term implementation within standard operating protocols. A context-aware and priority-aligned approach prevents unrealistic expectations leading to tailored, locally grounded plans that strengthen, rather than burden, ongoing diagnostic and surveillance activities.

### Rule 2: Anticipate and overcome infrastructure challenges

Regions with limited infrastructure often face barriers such as restricted internet connectivity, unstable power supply, or the lack of secure computational servers. Anticipating these challenges is critical for ensuring training continuity and long-term sustainability.

Before training begins, one needs to validate equipment functionality, confirm that sequencing instruments are properly serviced, and ensure that computers meet minimum software and security requirements. When on-site resources are insufficient, leverage cloud-based platforms or partner institutions with established infrastructure to host training sessions or computational pipelines. Transitioning from cloud-based training platforms to institutional and laboratory infrastructure should be prioritized, with permission for remote access obtained in advance. This should preferentially also include a plan for in-person and on-site support (e.g., weekly visits by a local trainer) for the periods when remote access is delayed or cannot be granted. The transition should also consider implementations and software availability that may differ between the training and production infrastructure, ensuring that trainees become familiar with a similar context in both environments. Developing backup plans, redundancy strategies, and technical support partnerships minimizes interruptions and guarantees equitable access to training resources across diverse regions.

The co-created standard operating procedures (SOPs) should incorporate these best practices to ensure secure data storage, privacy, and controlled access through encryption and appropriate access restrictions, safeguarding sensitive health information in compliance with national legislation. Standardizing and containerizing bioinformatics workflows (e.g., Docker or Singularity) during training should ensure that the same validated analysis environment can be deployed consistently across laboratories, regardless of their underlying hardware. These containerized workflows, together with workflow management systems such as Nextflow and Snakemake, should be documented within the SOPs (Rule 5), to promote reproducibility, portability, and consistent implementation across participating laboratories.

### Rule 3: Plan and procure well in advance

Procurement is a recurrent bottleneck in implementing genomics in public health laboratories. To minimize delays, institutions should establish procurement and infrastructure preparation timelines synchronized with training schedules, accounting for local bureaucratic processes, customs clearance, reagent shelf life, and transport logistics. Planning should also include establishing the infrastructure that will enable training, such as ensuring dedicated laboratory space, stable power supply, reliable internet connectivity, functional biosafety equipment, and computers capable of running bioinformatics analyses (at minimum, a multi-core processor, 16–32 GB of RAM, and 512 GB up to 2 TB of storage, ideally complemented by access to a dedicated server or cloud-computing resources for larger or production-scale workloads; this applies both to computers used during training and, more importantly, to those used afterwards in routine scientific or diagnostic practice). Installing and validating these resources before the training begins allows participants to practice on real systems and facilitates a smoother transition to routine implementation afterwards. Planning should also involve coordinating with administrative and management teams to secure time release for trainees and ensure institutional support. A rolling procurement plan (S1 Table) that is shared with transparency and includes reagents and consumables (with supplier details), small equipment, and shipping details to prepare for training phases helps maintain continuity and improves coordination between technical and administrative staff. Securing joint financial support from ministries, research institutions, and funding agencies also strengthens sustainability and institutional commitment.

### Rule 4: Design training as a continuous cycle

Capacity building should be envisioned as a continuous process, not a once-off event. A cyclical model combines (i) workshops and webinars; (ii) in-person practical sessions to establish foundational skills; (iii) regular virtual living follow-ups (weekly, biweekly, and monthly) to reinforce learning, troubleshoot challenges, and adapt to new technologies; (iv) pre-recorded online sessions; and, (v) pre-, during and post-training evaluations to assess progress and inform future activities. It is imperative to highlight that the content should (also) be delivered in the local language to maximize understanding and access to the training material. Translation is best approached by involving bilingual co-trainers to validate technical terminology, maintaining a shared glossary of standardized terms across languages, and prioritizing the translation of the most frequently used materials, such as SOPs and checklists, ahead of less time-critical content such as slide decks.

This cyclical structure is conceptually aligned with the Bicycle Principles framework for short-format training [10], which organizes recommendations around two iterative cycles: (i) a core cycle grounded in best evidence, effectiveness, and inclusion, and (ii) a community cycle oriented towards reach, scale, and sustainability. Embedding the Bicycle Principles’ emphasis on catalytic learning, a learning that equips participants to continue learning independently after formal training ends and ensures that periodic refreshers and peer mentoring translate short training events into lasting institutional capability, consistent with Rules 6–9 below.

This iterative approach transforms workshops into learning ecosystems where participants gain confidence and sustain engagement over time. Continuous mentoring and periodic refresher sessions help consolidate knowledge, reduce skill decay, and enable participants to evolve from trainees into trainers, multiplying local expertise and strengthening institutional autonomy. In addition, regular quality assurance, proficiency testing, and performance evaluations, conducted monthly, are essential for maintaining consistency and ensuring that technical skills remain updated and reliable.

### Rule 5: Co-create laboratory-specific protocols and checklists

Each laboratory operates within a unique context, including personnel, infrastructure, and workflows. To ensure reproducibility and standardization, it is essential to co-develop SOPs, workflows, and checklists tailored to local conditions. Co-creation of these documents by trainers, laboratory teams, and quality assurance officers ensures that protocols are both practical and owned by those who will apply them daily.

This participatory process promotes accountability and encourages laboratories to embed quality assurance practices, from sample extraction and quality control to bioinformatics analysis. Visual SOPs and step-by-step checklists can enhance comprehension and adoption across diverse technical backgrounds, especially when supported by graphics and translated into local languages. Documenting these processes also supports inter-laboratory comparisons, standardization, and continuous improvement within national or regional networks.

### Rule 6: Encourage shared ownership and co-create educational materials for sustainability

Empowerment is key to sustainability. Encouraging participants to take ownership of their learning and contribute to the creation of training materials fosters engagement and inclusion. Trainees can collaborate on developing or refining SOPs, troubleshooting guides, and teaching slides, adapting language and examples to their own laboratory realities. This generates involvement during the full project lifecycle: starting with project design, over training delivery, when trainees and trainers jointly edit shared materials, contributing figures, examples, and troubleshooting fixes to shared code; until after training, when accumulated field experience is incorporated into finalized, peer-reviewed versions of the materials.

Inviting participants and trainers to act as co-developers or co-authors of educational materials not only enhances visibility but also ensures that content evolves organically with technological updates and field experiences. Co-creation promotes a sense of authorship, equity, and diversity, transforming participants from passive learners into active contributors, reinforcing the long-term sustainability of the training ecosystem. Formalizing institutional commitments and governance structures, through cooperation agreements, policies, or joint authorship frameworks, further embeds genomics into institutional memory and secures its continuation beyond individual projects.

### Rule 7: Share training materials and resources in a centralized platform

Maintaining training materials in a centralized, accessible platform ensures consistency, equity, and long-term usability. All SOPs, manuals, videos, presentations, and evaluation forms should be stored in a shared repository that enables asynchronous learning and continuous feedback. Bioinformatics workflows and their associated code, scripts, and containerized computational environments (e.g., Docker or Singularity images, or pre-configured virtual machines) used during training should be deposited in the same repository (e.g., GitHub, GitLab, or BitBucket), so that other laboratories can reproduce not only the training curriculum, but also the underlying analytical pipelines. Digital learning environments such as Google Classroom or institutional intranets can serve as hubs for collaborative learning and documentation. Recorded virtual sessions, unlisted video links on public video-sharing platforms such as YouTube, and downloadable materials allow participants to revisit content at their own pace. When accompanied by standardized templates and naming conventions, centralized repositories also promote data integrity and interoperability across institutions.

### Rule 8: Expand expertise without redefining roles

Laboratory technicians need to understand the principles of bioinformatics, but they do not need to become full-time computational specialists. Likewise, bioinformaticians benefit from learning basic laboratory processes to contextualize data quality.

Identifying the focal points in each laboratory, individuals who bridge disciplines and support peer learning, helps strengthen teamwork and communication. Training opportunities should be aligned with participants’ professional interests and proficiency levels, ensuring depth of expertise where desired and literacy where needed. This mutual understanding promotes efficiency, respect, and smooth integration of genomics in existing workflows. Encouraging diversity in expertise also helps institutions distribute responsibilities equitably and avoid burnout among highly skilled staff.

### Rule 9: Foster communities and subcommunities of practice

Capacity building thrives within active and collaborative communities. Beyond formal training, laboratories benefit from creating communication channels and subcommunities of practice, for example, regional groups or thematic clusters (wet-lab, bioinformatics, and epidemiology).

Popular communication platforms such as groups on an instant-messaging platform, file-sharing platforms, and monthly virtual meetings facilitate real-time troubleshooting, peer mentorship, and collective problem-solving. These communities foster belonging, continuous engagement, and cross-institutional collaboration. They also help bridge disparities across regions, enabling laboratories facing similar challenges to share local solutions and promote equity in capacity development. Recognizing collective achievements and celebrating milestones strengthens motivation, reinforces identity, and sustains participation in the long term.

### Rule 10: Synchronize databases and promote cross-environment networking with public health authorities

Genomic data gains value when contextualized within clinical, epidemiological, and phenotypic frameworks. To enable this integration, laboratories should ensure communication between teams generating sequencing, diagnostic, and surveillance data. This might optimize joint efforts and contribute to harmonizing practices at a regional, national and/or supranational level. Establishing secure and interoperable databases supports information exchange and maximizes the usefulness of generated data.

Whereas the integration with national health information systems is still evolving across various countries in the Global South, creating secure, protected repositories (e.g., REDCap), allows laboratories to manage data responsibly while maintaining accessibility for clinical and public health interpretation. Harmonizing data formats, metadata standards, and bioinformatics pipelines across laboratories enhances interoperability and facilitates national data integration. Synchronizing databases and promoting networking between laboratory, clinical environments and other similar research initiatives strengthens data-driven decision-making and paves the way for the formal incorporation of genomics into health systems. Establishing clear governance mechanisms and defining roles, responsibilities, and equitable participation hopefully ensures that genomic networks operate transparently, collaboratively, and sustainably.

## Conclusion

Capacitating genomic-aided vigilance of transmissible diseases for routine laboratory use in the Global South is both a scientific and institutional endeavor and requires long-term planning, including training, and robust network formation connecting laboratories, academia, and public health systems. With our proposal of ten simple rules, we expect to emphasize that a co-creative, community-driven, and context-sensitive approach, one that recognizes that technology alone does not build capacity; people do.

Importantly, sustainability of such a society depends on creation of adequate human resources and their well-defined roles. Ideally, laboratories implementing genomic workflows should have at least one full-time bioinformatician dedicated to data analysis, management, and troubleshooting. This role is crucial for maintaining workflow continuity, ensuring data quality, and translating results into actionable insights.

However, frequent staff rotation and the multitasking nature of many laboratory positions remain persistent challenges to sustaining capacity. When trained personnel are reassigned or their time is split between multiple duties, institutional efficiency and technical continuity are disrupted. Addressing these issues through retention strategies, career incentives, and institutional commitments is essential for maintaining long-term genomic capability.

Beyond staffing and procurement, sustaining genomics capacity also requires anticipating equipment maintenance and service contracts, generally overlooked until instrument failure, alongside data-governance frameworks that define who may access, transfer, or interpret genomic data, besides accreditation or quality-assurance requirements that increasingly apply as laboratories move genomics into routine clinical or public-health use. Addressing these dimensions alongside human-resource retention strengthens the overall resilience of genomics programs.

By embedding genomics within local realities, securing dedicated expertise, and fostering collaboration across environments, laboratories can move from pilot initiatives to sustainable systems that contribute directly to public health action and global genomic equity. Taken together, the ten rules describe a single continuous pathway: diagnosing context and preparing infrastructure (Rules 1–3), building and transferring skills through iterative, co-created training (Rules 4–6), embedding those skills within sustained communities and shared infrastructure (Rules 7–9), and finally connecting the resulting genomic data to clinical, epidemiological, and policy decision-making (Rule 10). No single rule is sufficient on its own; it is their combined and sequential application that transforms genomics from a research activity into routine public-health practice.

## Supporting information

S1 Table Suggested initial checklist to guide procurement and planning. (XLSX)

S2 Table Checklist for computational training, covering trainee profiles, technical infrastructure, and training design. (XLSX)

S3 Table Checklist for sequencing instrument validation, covering equipment specifications, location, and validation status prior to routine use. (XLSX)
